# Parental health literacy and health knowledge, behaviours and outcomes in children: a cross-sectional survey

**DOI:** 10.1186/s12889-020-08881-5

**Published:** 2020-07-13

**Authors:** Elke de Buhr, Antje Tannen

**Affiliations:** 1 Charité – Universitätsmedizin Berlin, corporate member of Freie Universität Berlin, Humboldt-Universität zu Berlin, and Berlin Institute of Health and Nursing Science, Augustenburger Platz 1, D-13353 Berlin, Germany; 2 Tulane University, School of Public Health and Tropical Medicine, 1440 Canal St., New Orleans, LA 70112 USA

**Keywords:** Health literacy, Parents, Schoolchildren, Health behaviour, Health outcomes

## Abstract

**Background:**

Health literacy (HL) is closely associated with leading health indicators such as engaging in healthy behaviours and experiencing a healthy social environment. Parents represent a critical subgroup among the adult population since they are not only responsible for their own health but also for the health of their children. Previous research suggests that parents with low HL are less likely to meet the preventive and health care needs of their children but there are gaps in the available information and there is not any data available yet for the German context.

**Methods:**

In preparation of an implementation study, a cross-sectional survey was conducted in 28 elementary and secondary schools in Germany. The parent questionnaire was completed by 4217 parents and included the short form of the European Health Literacy Survey Questionnaire (HLS-EU-Q16). The child questionnaire examined children’s health knowledge, behaviours and outcomes. For children between 6 and 10 years, a parent reported on behalf of their children (*N* = 1518). Students 11 years and older completed a self-administered questionnaire (*N* = 2776). Bivariate and multivariate analyses were carried out. Spearman’s Rho correlations assess the relationships between household characteristics, parental HL and the health behaviour and outcomes in children.

**Results:**

Among the participating parents, 45.8% showed problematic or inadequate HL. The major determinants of high parental HL were high socio-economic status (SES) (r = .088***, 95% CI [.052, .124]), living in West Germany (r = .064***, 95% CI [.032, .096]) and older parental age (r = .057**, 95% CI [.024, .090]). In the multivariate model, only SES remained significant. High parental HL was associated with positive health behaviours in children including healthier nutrition, regular tooth brushing and more physical activity. The relationships between parental HL and smoking, alcohol, sexual activity among children and children’s weight were not significant.

**Conclusions:**

The results confirm a relationship between low parental HL, SES and some child health behaviours likely to negatively impact their health and wellbeing including less healthy nutrition and less exercise. Strengthening the health knowledge and competencies of parents may contribute to improved child outcomes particularly in the areas of nutrition, exercise and dental health.

## Background

Among the leading health indicators are engaging in healthy behaviours, experiencing a healthy social environment, engaging in responsible sexual behaviour, engaging in substance abuse and using tobacco [1]. Each of these indicators depends to some extent on the information people have about their health and how they use it (knowledge aspects), health-related choices (behavioural factors), the socio-economic and environmental context (environmental, economic and social conditions) as well as the type, quantity and quality of health care people receive (system related factors) [1]. The concept of health literacy is used in various definitions and has been measured with numerous instruments. There is agreement, however, that poorer health literacy is associated with poorer health knowledge, more problematic health behaviours, worse health outcomes, inadequate use of health services and increased health care costs [2, 3].

Parents with young children represent a critical subgroup among the general adult population since they are not only responsible for their own life and health but as parents and caregivers they are also responsible for the health and wellbeing of their children. Children are dependent on their parents to prevent and address health problems and they may suffer when their parents’ knowledge and skills to fulfil these tasks is insufficient [4, 5]. A study by Sanders et al. [6] describes parental literacy as an important moderator of child health disparities. The research establishes relationships between low parental health literacy and unmet health care needs of children, unnecessary visits to emergency departments, ineffective injury prevention, mistakes in the administration of medication, poor childhood nutrition, inaccurate perception of children’s weight (overweight children are perceived as normal weight or underweight) as well as increased risk of exposure to second hand tobacco smoke [6]. A systematic review by DeWalt [7] suggests that poorer parental health literacy is associated with poorer knowledge about health outcomes, behaviours and health services, higher rates of hospitalisations and emergency room visits in children with asthma as well as increased use of non-standardised dosing instruments for administering liquid drugs. A systematic review by Firmino [8] focused on parental oral health literacy and found associations between lower parental oral health literacy and higher rates of dental caries in children as well as poorer oral health-related quality of life [8]. The most recent systematic review by Morrison et al. [5] confirms some of the earlier findings, including the association between parental health literacy and child health but in other areas research findings are inconsistent (e.g., previous findings regarding preventive care, acute care, chronic diseases, the effectiveness of interventions). Drawing on this emerging evidence, multiple recent intervention studies have focused on parental health literacy as part of the intervention, e.g. to treat childhood obesity [9] or to reduce medication errors [10].

A comparison of health literacy survey data from eight European countries shows that adults frequently have difficulties with health-related information such as understanding how political decisions affect health outcomes, judging the credibility of health information in the media or deciding between different treatment options. The prevalence of problematic or inadequate health literacy in the eight countries participating in the survey ranged from 29 to 62% of the population. In addition, health literacy disparities were observed within each studied population. On average, health literacy was higher for young people with low financial deprivation, for individuals with higher social status and education as well as for women. The level of individual health literacy also was associated with level of physical activity, body mass index (BMI) and alcohol consumption [11].

Although health literacy is receiving increasing attention on the German political stage [12], the available evidence base is still limited. As part of the first round of European health literacy surveys, only one German state (North Rhine-Westphalia) participated. In this German subsample the prevalence of problematic or inadequate health literacy was found to be 46.3%. The survey confirmed relationships between health literacy and smoking as well as between health literacy and physical exercising. However, significant associations could not be established between health literacy and alcohol consumption as well as between health literacy and BMI [11]. The research did not examine the relationship between parental health literacy and child health behaviours or outcomes.

The study presented in this article addresses some of the gaps in the German health literacy research field and may serve to further validate previous findings on the relationship between parental health literacy and child health behaviour and outcomes. It provides the first data to quantify the level of parental health literacy in Germany. In addition, the social determinates of parental health literacy are analysed and vulnerable subgroups of adults and children are identified. We expect that the research findings may contribute to developing and improving health literacy and health information campaigns and other social interventions targeted at parents and their school-aged children.

## Methods

The aim of this study was to better understand parental health literacy in Germany and assess the implications for children’s health. Specific objectives were directed at quantifying the level of health literacy among parents of school-aged children including their ability to find, understand and appraise health information. The research also aimed to identify health-related skills that are perceived as challenging and document disparities in parental health literacy based on socio-demographic factors. The research finally intended to document the implications of parental health literacy for children’s health behaviours and outcomes.

A large cross-sectional survey was carried out in preparation of an implementation study, which placed school nurses at 28 public schools in two federal states in Germany, Brandenburg and Hessen, that did not previously have access to this resource [13–15]. The elementary and secondary schools were selected by the local governments using a purposeful selection process. The 2017 baseline data collection was directed at examining and quantifying relevant health-related conditions. Primary and secondary school systems in Germany differ by state. In Hessen primary schools cover grades 1–4, in Brandenburg they cover grades 1–6. Subsequently, students enter different types of secondary schools and graduate either after grade 10 (vocational track) or after grade 12 (university track). All parents with children in the selected schools and all children 11 years and older attending the schools were invited to participate in the survey. Children and adults received ID numbers to allow the pairing of responses.

The parent questionnaire was completed by 4217 parents. It covered household socio-demographic, health and general wellbeing indicators. It also included the short form of the European Health Literacy Survey Questionnaire (HLS-EU-Q16). Based on a conceptual model of health literacy by Sorensen et al. [16], the original HLS-EU-Q47 was developed by the HLS-EU Consortium. The integrative model addresses the competencies of accessing, understanding, appraising and applying health-related information in the areas of health promotion, disease prevention and healthcare [16]. The reliability of the scale’s 47 self-reported single items was found acceptable for the German version and reached Cronbach’s Alpha coefficients between 0.78 and 0.93 [17]. The HLS-EU-Q16 Short Scale, which was applied in this study, was derived using iterative item selection based on Rasch modeling (1-parametric dichotomous model) as well as content and face validity criteria [18]. The HLS-EU-Q16 scores were transformed into a unified metric scale with a minimum of 0 and a maximum of 50 points. The minimum of 0 points represents the least possible parental health literacy and the maximum of 50 points represents the highest possible score [11, 17, 19]. To assess socio-economic status (SES) and child health behaviours, standardized tools from large epidemiological surveys were adapted [20–22]. The SES of households was measured using a combination of items: Educational level, vocational training level, current job and income.

The child questionnaire, adapting questionnaire items from previous epidemiological surveys [20–22], covered health knowledge, behaviours and outcomes including nutrition [22], physical activity [21], dental care [21], smoking [20], alcohol use [21], sexual activity [22] as well as BMI [20]. If the child was between 6 (age of school entry) and 10 years, the parent reported on behalf of their children (*N* = 1518). For children 11 years and older, the children completed the questionnaires, which in addition assessed smoking, alcohol use and sexual activity, themselves (*N* = 2776). The minimum age of 11 years for the child questionnaire was selected based on the reading skills and other cognitive competencies needed to complete the form. The questionnaires were self-administered and a pilot test involving a small number of children was completed before the start of data collection to obtain feedback on item comprehension and time needed to complete the form. Completing the questionnaire was voluntary and all data were kept confidential, and an informed consent process was followed. All participants were informed about the objectives of the study and its role in the context of the school nursing intervention. No incentives were offered to parents or children for participation in the research. The parents completed their questionnaires at home, and their children returned these questionnaires in closed envelopes to the school. If parents had more than one child enrolled at the school, they reported on all of their children. The participating children completed their questionnaires in class during a period of time set aside for this task. The completed questionnaires were scanned and entered into an electronic database. The levels of measurements were nominal (sexual activity), ordinal (physical activity, dental care, nutrition, alcohol consumption, smoking, SES) or interval-ratio (BMI, health literacy). Bivariate and multivariate analyses were carried out. Spearman’s Rho correlations assess the relationships between parental health literacy, household demographic and socio-economic characteristics, and the health behaviour and outcomes in children.

## Results

### Socio-demographic characteristics

Among the participating parents were 3259 mothers (77.1%), 675 fathers (16.0%) as well as a minority of 44 “other parents” (1.0%), for example, grandparents. An additional 248 parents (5.9%) declined to provide information on their relationship to the child. The age of the parents ranged from 19 to 76 years with an average age of 38.1 years withhe vast majority of parents between 25 and 55 years old. The 2773 participating children were on average 14.2 years old at the time of data collection with 51.7% of the children female and 48.3% male (see Table 1).

**Table 1:** Demographic Characteristics of Samples

**Average Age of Parents**	38.1 years
**Average Age of Children**	14.2 years
**Gender of Parents**	Female: 77.1%
	Male: 16.0%
	Other: 1.0%
	No response: 5.9%
**Gender of Children**	Female: 51.7%
	Male: 48.3%
**Number of Children**	One child: 24.9%
	Two children: 45.0%
	Three children: 17.5%
	Four children: 6.1%
	Five children: 1.5%
	No response: 5.1%
**Child with Chronic Condition or Disability**	8.4%
**Urban/Rural Location**	Above 100,000: 32.7%
	20,000–100,000: 37.3%
	10,000–20,000: 21.7%
	Under 10,000: 8.3%
**German State**	Brandenburg (East Germany): 54%
	Hessen (West Germany): 46%
**N (Parents)**	**3980**
**N (Children)**	**2773**

Families with two children were most common among the interviewed households. Among the survey respondents, 1052 parents reported having one child (24.9%), 1901 parents reported two children (45.0%), 738 parents reported three children (17.5%), 256 parents reported four children (6.1%) and 64 parents reported five children (1.5%). An additional 251 parents declined to provide information on the number of their children (5.1%). While most parents did not report caring for a child with a health problem, 353 parents (8.4%) indicated that their child had a chronic condition or disability (see Table 1).

In terms of socio-economic status, the participants in the survey varied widely. Among the 3189 households that provided the required information on educational level, vocational training, job position, and income, 1047 households (32.8%) had a high socio-economic status, 1358 households (42.6%) had a medium socio-economic status and 784 households (24.6%) had a low socio-economic status (see Table 1).

Both urban and rural schools were represented in the sample with 32.7% of the interviewed children attending school in cities above 100,000 inhabitants, 37.3% attending school in cities between 20,000 and 100,000 inhabitants and 21.7% attending school in towns between 10,000 and 20,000 inhabitants. Less than 10% of the children attended schools in communities with fewer than 10,000 inhabitants. West German households were represented with 54.0% of the sample and East German households with 46.0% (see Table 1).

The survey population, while not a representative sample and limited to two German states, closely mirrors the population of school-aged children and their parents in Germany with regard to age, sex and social status. In terms of migration status, children with migration background also resembled the German population with 37.4% of the children in the sample with this characteristic compared to 36% of the children in the general population [23].

### Results for single items of parental health literacy

Finding “information on how to manage mental health problems like stress or depression” was judged to be “very difficult” by 6.4% of the reponding parents and “fairly difficult” by 34.8%. Judging “if the information on health risks in the media is reliable” was rated as “very difficult” by 6.2% of respondents and as “fairly difficult” by 44.0%. Also perceived as difficult was judging “when you may need to get a second opinion from another doctor” with 3.9% of respondents considering this task to be “very difficult” and 32.5% “fairly difficult.” Similarly, deciding “how you can protect yourself from illness based on information in the media” was experienced as challenging with 3.9% of the respondents reporting that this task was “very difficult” and 35.8% reporting it was “fairly difficult” (see Table 2).

**Table 2:** HLS-EU-Q16 Health Literacy Matrix Items for Sample

Item	On a scale from very easy to very difficult, how easy would you say it is to ...	very difficult	fairly difficult	fairly easy	very easy	N
1	find information on treatments of illnesses that concern you?	1.7%	22.2%	59.4%	16.7%	3813
2	find out where to get professional help when you are ill?	1.6%	19.0%	55.2%	24.3%	3882
3	understand what your doctor says to you?	1.3%	13.8%	57.6%	27.4%	3920
4	understand your doctor’s or pharmacist’s instruction on how to take a prescribed medicine?	0.4%	3.1%	43.6%	52.9%	3943
5	judge when you may need to get a second opinion from another doctor?	3.9%	32.5%	45.2%	18.4%	3887
6	use information the doctor gives you to make decisions about your illness?	1.8%	25.3%	56.6%	16.4%	3853
7	follow instructions from your doctor or pharmacist?	0.5%	4.3%	54.7%	40.6%	3921
8	find information on how to manage mental health problems like stress or depression?	6.4%	34.8%	44.5%	14.3%	3731
9	understand health warnings about behaviour such as smoking, low physical activity and drinking too much?	1.2%	8.0%	44.3%	46.4%	3849
10	understand why you need health screenings?	0.6%	5.1%	40.9%	53.4%	3895
11	judge if the information on health risks in the media is reliable?	6.2%	44.0%	38.7%	11.1%	3854
12	decide how you can protect yourself from illness based on information in the media?	3.9%	35.8%	46.8%	13.5%	3845
13	find out about activities that are good for your mental well-being?	2.6%	28.9%	52.7%	15.8%	3781
14	understand advice on health from family members or friends?	1.0%	10.6%	55.5%	32.9%	3849
15	understand information in the media on how to get healthier?	2.0%	19.1%	57.6%	21.3%	3827
16	judge which everyday behaviour is related to your health?	1.5%	15.9%	56.9%	25.8%	3845

On the positive side, following “instructions from your doctor or pharmacist”, following “your doctor’s or pharmacist’s instruction on how to take a prescribed medicine”, understanding “health warnings about behaviour such as smoking, low physical activity and drinking too much” and understanding “why you need health screenings” were perceived as "very easy" or "fairly easy" by most of the respondents with less than 10% of respondents considering these tasks to be “very difficult” or “fairly difficult” (see Table 2).

### Prevalence of categories of parental health literacy in the sample

Among the 3980 parents who provided the required information, 14.9% had excellent health literacy, 39.3% had sufficient health literacy, 33.7% had problematic health literacy, and 12.1% had inadequate health literacy (see Table 3).

**Table 3:** Health Literacy Categories for Sample^a^

	Frequency	Percent
Excellent	592	14.9
Sufficient	1564	39.3
Problematic	1342	33.7
Inadequate	482	12.1
Total	3980	100

^a^*Calculated using the HLS-EU methodology* [11] *using the following scores: Inadequate health literacy ranging from 0 to 25, problematic health literacy ranging from > 25–33, sufficient health literacy ranging from > 33–42 and excellent health literacy ranging from > 42–50*

### Social determinants of parental health literacy

The major determinants of high parental health literacy were high socio-economic status (SES) (r = .088***, 95% CI [.052, .124]), living in West Germany (r = .064***, 95% CI [.032, .096]) and older parental age (r = .057**, 95% CI [.024, .090]). None of the other socio-demographic variables (“Gender of Parent”, “Number of Children”, “German Native Speaker”, “Child with Chronic Condition or Disability” and “Rural/Urban Location”) was a significant determinant of health literacy (see Table 4).

**Table 4:** Demographic Characteristics and Parental Health Literacy

Parental Health Literacy and …	r	95% CI	N
Gender of Parent (1 = male, 2 = female)	.021	−.012, .054	3454
Age of Parent (in years)	.057^b^	.024, .09	3388
Number of Children	−.032	−.064, .000	3691
German Native Speaker	.022	−.01, .054	3612
Child with Chronic Condition or Disability	−.013	−.045, .019	3568
Socio-Economic Status of Household (based on parents’ education and income)	.088^c^	.052, .124	2844
Rural/Urban Location (1 = urban, 5 = rural)	−.025	−.057, .007	3684
East/West German Location (0 = East German, 1 = West German)	.064^c^	.032, .096	3691

^*a*^*Spearman’s Rho Correlation, significant at the .05 level (2-tailed)*

^*b*^*Spearman’s Rho Correlation, significant at the .01 level (2-tailed)*

^*c*^*Spearman’s Rho Correlation, significant at the .001 level (2-tailed)*

Further analysis revealed moderate inter-correlations between the three significant predictors of parental health literacy: “Socio-Economic Status of Household”, “East/West German Location” and “Age of Parent” (see Table 5).

**Table 5:** Correlations between Age of Parent, Socio-Economic Status and East/West German Location

		East/West German Location	Age of Parent	SES of Household
**East/West German Location**	**Correlation Coefficient** **N**	1	.429^c^	.362^c^
		3021	3021	3021
**Age of Parent**	**Correlation Coefficient** **N**	.429^c^	1	.372^c^
		3021	3021	3021
**SES of Household**	**Correlation Coefficient** **N**	.362^c^	.372^c^	1
		3021	3021	3021

^*a*^*Spearman’s Rho Correlation, significant at the .05 level (2-tailed)*

^*b*^*Spearman’s Rho Correlation, significant at the .01 level (2-tailed)*

^*c*^*Spearman’s Rho Correlation, significant at the .001 level (2-tailed)*

Multivariate analysis points to SES as the most important socio-demographic factor explaining differences in health literacy in the survey population and the only significant predictor in the multiple regression models (see Table 6). After excluding the non-significant predictor age (in Model 2) as well as age and location (in Model 3), SES further increases in significance while the explained variance remains equal. However, the models only explain a small percentage of the total variation of health literacy among parents.

**Table 6:** Multiple Regression Model Predicting Variation in Parental Health Literacy

Independent Variables^**a**^	Dependent Variable – Parental Health Literacy		
	Model 1	Model 2	Model 3
**Intercept**	33.495***	78.650***	78.660***
**East/West German Location**	.643	.769	–
**Age of Parent**	.322	–	–
**SES of Household**	4.327***	4.660***	5.284***
**Model Fit**			
**R**^**2**^	.010	.010	.010
**Adjusted R**^**2 b**^	.009	.009	.009
**F**	9.236***	14.254***	27.921***
**N**	2682	2843	2843

*p < .05, **p < .01, ***p < .001

^**a**^*Entries are regression coefficients*

^**b**^*The Adjusted R*^*2*^*adjusts for number of variables relative to number of cases*

The data also indicate that there is a relationship between parental health literacy and self-reported health and wellbeing. Parents with high health literacy reported a better subjective health (r = .111***, 95% CI [.079, .143]) and quality of life (r = .136***, 95% CI [.104, .168]) (see Table 7).

**Table 7:** Parental Health Literacy and Subjective Health and Life Satisfaction

Parental Health Literacy and …	r	95% CI	N
Subjective Health of Parents	.111^c^	.079, .143	3468
Life Satisfaction of Parents	.136^c^	.104, .168	3435

^*a*^*Spearman’s Rho Correlation, significant at the .05 level (2-tailed)*

^*b*^*Spearman’s Rho Correlation, significant at the .01 level (2-tailed)*

^*c*^*Spearman’s Rho Correlation, significant at the .001 level (2-tailed)*

### Parental health literacy and health outcomes in children

Based on the analysed data, there are strong statistical relationships between parental health literacy and a number of health behaviours in children. Children under 11 years who had parents with high health literacy were eating more vegetables and salad (r = .100***, 95% CI [.047, .153]) and more fruits (r = .086**, 95% CI [.032, .139]) than other children. These children were more frequently engaged in physical activity (r = .079**, 95% CI [.025, .132]), they were more likely to brush their teeth regularly (r = .055*, 95% CI [.001, .108]), and their parents judged them as healthier (r = .144***, 95% CI [.091, .196]). Relationships between parental health literacy and children’s BMI as well as their days missed at school in the last term could not be established (see Table 8).

**Table 8:** Parental Health Literacy and Adult-Reported Health Behaviours and Outcomes for Children 6 to 11 Years

Parental Health Literacy and …	r	95% CI	N
Children’s Regular Physical Activity	.079^b^	.025, .132	1299
Children’s Regular Tooth Brushing	.055^a^	.001, .108	1306
Children’s Regular Visit to Dentist	.020	−.034, .074	1294
Children’s Healthy Nutrition – Consumption of...			
Fruit	.086^b^	.032, .139	1310
Vegetables, Salad	.100^c^	.047, .153	1310
Chocolate and other Sweets	.037	−.017, .090	1310
Potato Chips, Fries and Similar	−.047	−.100, .007	1310
Cola and Other Sweetened Beverages	.009	−.045, .063	1310
Children’s BMI	.002	−.057, .061	1086
Children’s Subjective Health	.144^c^	.091, .196	1298
Children’s Number of Days Missed at School	−.054	−.111, .004	1131

^*a*^*Spearman’s Rho Correlation, significant at the .05 level (2-tailed)*

^*b*^*Spearman’s Rho Correlation, significant at the .01 level (2-tailed)*

^*c*^*Spearman’s Rho Correlation, significant at the .001 level (2-tailed)*

In the older age group (11+ years), children of parents with better health literacy reported eating more vegetables and salad (r = .163*, 95% CI [.115, .211]), and they were drinking fewer sweetened beverages (r = −.096***, 95% CI [−.145, −.047]). They also reported brushing their teeth more regularly (r = .058*, 95% CI [.008, .107]). However, there were no significant relationships between parental health literacy and indicators measuring physical activity, alcohol and tobacco consumption, and unprotected sexual activity in this age group. There also were no significant relationships between parental health literacy and children’s BMI, child-reported subjective health or their days missed in school in the last term (see Table 9).

**Table 9:** Parental Health Literacy and Child-Reported Health Behaviours and Outcomes for Children 11 Years and Older

Parental Health Literacy and …	r	95% CI	N
Children’s Regular Physical Activity	.001	−.06, .062	1001
Children’s Regular Tooth Brushing	.058^a^	.008, .107	1526
Children’s Regular Visit to Dentist	.007	−.043, .057	1506
Children’s Healthy Nutrition – Consumption of...			
Fruit	.037	−.012, .086	1551
Vegetables, Salad	.163^a^	.115, .211	1551
Chocolate and other Sweets	−.007	−.056, .042	1551
Potato Chips, Fries and Similar	−.040	−.089, .009	1551
Cola and Other Sweetened Beverages	−.096^c^	−.145, −.047	1551
Children’s Alcohol Consumption	.005	−.046, .056	1476
Children’s Tobacco Consumption	−.013	−.063, .037	1498
Children’s Sexual Activity without Protection	−.020	−.072, .032	1408

^*a*^*Spearman’s Rho Correlation, significant at the .05 level (2-tailed)*

^*b*^*Spearman’s Rho Correlation, significant at the .01 level (2-tailed)*

^*c*^*Spearman’s Rho Correlation, significant at the .001 level (2-tailed)*

## Discussion

Among the participating parents 45.8% showed a problematic or inadequate health literacy. This percentage is comparable with the results from the first German-wide health literacy survey. In the 2016 survey of the general population, problematic or inadequate health literacy was reported by 41.4% in the area of coping with disease, 47.7% in the area of disease prevention, and 60.3% in the area of health promotion [17]. The health literacy scale’s single items indicated that media literacy was a particular problem for many parents (“judge information on health risks in the media”, “protect yourself from illness based on information in the media”), judgement calls were also difficult and information on mental health and treatments were perceived as insufficient. On the other hand, understanding health professionals and following basic instructions were regarded as comparably easy. This may point to information overload and online misinformation as possible factors contributing to problems with accessing and using the available health information. In the German wide health literacy survey, difficulties with media information were reported by 49.3% of the participants [17].

SES was the best predictor of health literacy in parents and the only socio-demographic variable that remained significant in the multivariate model. The correlations between parental health literacy and the subjective health of parents as well as parental health literacy and the quality of life of parents confirm that individuals with higher health literacy (and higher SES) also tend to have better subjective health and quality of life. While the correlation between SES and health literacy are in line with previous empirical research [11] and with theoretical models of health literacy [12, 16], the explanatory power of SES as a predictor of health literacy was limited. Besides personal skills and abilities, including the knowledge, motivation and competences to deal with health information, the demands of the needed evidence as well as the perceived and actual complexities of the health system also exert an influence when explaining self-perceived health literacy [16].

Confirming earlier studies, the research showed a relationship between the health and wellbeing of children and the health literacy of their parents. High parental health literacy was associated with positive health behaviours in children including higher consumption of fruits and vegetables, less consumption of sweetened beverages, regular tooth brushing as well as more frequent physical activity. The relationships between parental health literacy and children’s smoking, children’s alcohol consumption and risky sexual activity among children (for children 11 years and older) as well as children’s BMI (both age groups), however, were not significant and other factors have to be considered.

The research provides the first quantitative data on the relationship between parental health literacy and the health behaviours of school-aged children in Germany. Among the strengths of the study are the large sample and the diversity of the participating schools, which approximate the complexity of the German educational landscape. The study also benefits from access to and the ability to compare responses from both parents and their children. Potential limitations include the non-probability selection of the schools, a regional distribution that does not cover all federal states and some survey/item non-response caused by the research’s voluntary nature. In addition, both adults and children may have experienced challenges with responding to the survey questions. Self-reporting on health literacy and other health-related topics is vulnerable to social desirability bias, perhaps even more so in the context of a school nursing intervention. It is also conceivable that the response behaviour of the participants was influenced by their opinion of the school nursing intervention.

## Conclusions

The results confirm a relationship between low parental health literacy, SES and some child health behaviours likely to negatively impact their health and wellbeing including less healthy nutrition and less exercise. Hence these factors should be monitored as vulnerable families may require additional support to improve their health knowledge, access the available health information, navigate the health care system and with health-related decisions. Strengthening the health knowledge and competencies of parents may contribute to improved child outcomes particularly in the areas of nutrition, exercise and dental health.

While the sample was not representative of the entire country, the collected data provide a first window into the relationship between parental health literacy and child health in Germany. Additional research is needed to further explore the relative contribution of parental health literacy to children’s health behaviours and outcomes.

## Data Availability

Due to data protection regulations, the data of the participating parents and children are confidential and cannot be shared for further analyses.

## Abbreviations

BMI: Body Mass Index
HL: Health Literacy
HLS-EU: Health Literacy Survey – European Union
SES: Socio-Economic Status
SPLASH: Schulgesundheitspflege an allgemeinbildenden Schulen/School Nursing at General Schools
US: United States of America
